# *Mycobacterium tuberculosis* and HIV Co-Infection: A Public Health Problem That Requires Ongoing Attention

**DOI:** 10.3390/v16091375

**Published:** 2024-08-29

**Authors:** Yinzhong Shen

**Affiliations:** Department of Infection and Immunity, Shanghai Public Health Clinical Center, Fudan University, Shanghai 201508, China; shenyinzhong@shphc.org.cn

According to the World Health Organization’s (WHO) 2023 Global Tuberculosis Report, in 2022, HIV-positive tuberculosis cases accounted for 6.3% of all new tuberculosis infections, with a total of 167,000 HIV-positive individuals succumbing to tuberculosis worldwide [1]. HIV and *Mycobacterium tuberculosis* (MTB) co-infection remains a significant public health challenge, impacting upon population health. In co-infected individuals, complex interactions occur between HIV and MTB, where HIV-induced immunodeficiency facilitates MTB infection, disease progression, and dissemination. Conversely, MTB promotes HIV replication and immune activation. HIV/MTB co-infected individuals typically exhibit higher HIV viral loads, larger viral reservoirs, more pronounced abnormal immune activation, and a greater prevalence of disseminated tuberculosis [2]. The pathogenesis and management of tuberculosis in individuals with HIV/AIDS have unique characteristics. A diagnosis of HIV/MTB co-infection is particularly challenging. Its treatment entails specific considerations involving both anti-tuberculosis therapy and antiretroviral therapy (ART), with drug adverse effects, adherence, and drug–drug interactions influencing the therapeutic outcomes. The success rate of tuberculosis treatment in people living with HIV/AIDS is relatively low, with the 2023 WHO report stating an 88% success rate for drug-susceptible TB patients in 2021 compared to only 79% for HIV-positive tuberculosis patients [1]. Thus, understanding and addressing the diagnosis and treatment of HIV/AIDS that is complicated by tuberculosis remain paramount.

In this Special Issue “Tuberculosis (TB) and HIV Coinfection”, we present five articles, including four original research papers and one review, primarily focusing on HIV’s impact on the pathogenesis and clinical management of HIV/MTB co-infection. MTB can lead to latent tuberculosis infection (LTBI) and active tuberculosis in HIV-infected individuals. Screening and intervention for LTBI in HIV-positive individuals are crucial for preventing active disease, as highlighted by a study underscoring the importance of enhanced LTBI screening and implementation [3]. The mechanisms through which HIV promotes tuberculosis dissemination are a recent research focus. One article explored the effects of HIV on tuberculous granuloma formation through clinical biopsy analyses, revealing reduced CD4+ T lymphocyte counts in both peripheral blood and granulomas, associating CD4+ T cell depletion with impaired granuloma integrity, which is a key factor in the increased dissemination seen in HIV-infected tuberculosis patients [4]. With rising concerns over drug-resistant tuberculosis in HIV-infected populations [5], another study underscored the significance of HIV infection and ART on the success of resistant tuberculosis treatment, emphasizing the importance of early ART initiation for improved patient outcomes [6]. Monitoring treatment efficacy in co-infected patients was also addressed, with a study detailing dynamic changes in matricellular proteins (MCPs) in the peripheral blood of HIV/MTB co-infected individuals undergoing anti-tuberculosis therapy, suggesting that these markers could aid in predicting treatment responses and managing complications related to chronic immune activation [7]. Lastly, a review article [8] has summarized the therapeutic advancements in HIV/MTB co-infection, discussing new developments in ART and anti-tuberculosis regimens, as well as the challenges of drug interactions and prospective novel therapies, including targeted immunotherapeutic strategies, though highlighting the need for further research.

The editors would like to thank all the authors for their contribution to this Special Issue. The interplay between HIV and MTB represents a critical scientific issue demanding sustained attention. Future efforts must continue to investigate the mechanisms and management of HIV/MTB co-infection, particularly through clinical studies, to refine treatment strategies for dual-infected patients and to mitigate their morbidity and mortality rates.
